# Adduction Manipulation of the Glenohumeral Joint versus Physiotherapy for Atraumatic Rotator Cuff Tears: A Randomized Controlled Trial

**DOI:** 10.3390/jcm12124167

**Published:** 2023-06-20

**Authors:** Hiroshi Karasuno, Junichiro Hamada, Yuichiro Yano, Hiroaki Tsutsui, Yoshihiro Hagiwara, Kazuhiro Endo, Takashi Saito

**Affiliations:** 1 Department of Physical Therapy, Josai International University, Togane 283-0002, Japan; karasuno@jiu.ac.jp; 2 Department of Orthopaedic Surgery, Kuwano Kyoritsu Hospital, Koriyama 963-8034, Japan; yuu-yano@dokkyomed.ac.jp; 3 Department of Orthopaedic Surgery, Fujigaoka Hospital, Showa University, Yokohama 227-0043, Japan; tsutsui@yk.rim.or.jp; 4 Anylom Co., Ltd., Tokyo 104-0061, Japan; hagi@med.tohoku.ac.jp; 5 Department of Rehabilitation, Kuwano Kyoritsu Hospital, Koriyama 963-8034, Japan; kz_endo1983@yahoo.co.jp; 6 Department of Rehabilitation, Ono Orthopedic Clinic, Utsunomiya 321-0954, Japan; acetakashi18@yahoo.co.jp

**Keywords:** atraumatic rotator cuff tear, glenohumeral joint, adduction restriction, adduction manipulation, physiotherapy

## Abstract

Background: Atraumatic rotator cuff tears (ARCTs) are frequently concomitant with adduction restriction of the glenohumeral joint (GHJ). Adduction manipulation (AM) removes the restriction and relieves pain. The present study aimed to investigate the clinical efficacy of AM versus physiotherapy (PT) in ARCTs. Methods: Eighty-eight patients with adduction restriction were allocated to the AM and PT groups (*n* = 44 per group). The glenohumeral adduction angle (GAA) was calculated using X-rays at the first and last follow-up appointments. We recorded pain severity (visual analog scale, VAS), flexion, abduction, external rotation (ER), internal rotation (IR), and American Shoulder and Elbow Society (ASES) and Constant scores at baseline and at 1-, 3-, 6-, and 12- month follow-ups. Results: Forty-three patients (23 males, average age 71.3 years) in the AM group and 41 (16 males, average age 70.7 years) in the PT group were consequently analyzed. At the 1-month follow-up, VAS, shoulder motion except ER, ASES and Constant scores were much better in the AM group than in the PT group, whereas those in the PT group improved gradually up to 12 months. At the final follow-up, flexion, abduction, and Constant score were significantly better in the AM group than in the PT group. The GAA at the initial and final examinations was −21.6° and −3.2°, respectively, in the AM group, and −21.1° and −14.4°, respectively, in the PT group. Conclusions: The AM procedure, which had better clinical efficacy than PT, is recommended as the first conservative treatment option for ARCTs.

## 1. Introduction

Rotator cuff tears can be categorized into traumatic and atraumatic rotator cuff tears (ARCTs). Traumatic rotator cuff tears occurring in younger individuals are acute tendon injuries and treated operatively. ARCTs are found in 21% of elderly people over 50 years old and are treated conservatively; fewer than 20% of cases need surgical treatment [1,2]. Degenerative changes to the rotator cuff tendons and unknown etiologies contribute to the occurrence of ARCTs. Interestingly, 65% of individuals with ARCTs are asymptomatic; the remainder present with shoulder pain and disability [1,3]. The mechanisms underlying the development of symptomatic versus asymptomatic ARCTs are not fully understood.

The symptoms and pathology of ARCTs vary widely and include motion and/or night pain, crepitus, joint contracture, impingement, muscle weakness, instability, pseudoparalysis, and cuff tear arthropathy [4,5,6,7]. Treatment options are classified as conservative and surgical. Conservative treatment includes the administration of non-steroidal anti-inflammatory drugs (NSAIDs), the intra-articular injection of corticosteroids, physiotherapy (PT) and exercise therapy; these are recognized as first-line treatment options for ARCTs. Surgical treatments such as arthroscopic rotator cuff repair, superior capsular reconstruction, and reverse shoulder arthroplasty are recommended for patients with traumatic rotator cuff tears, muscle weakness, instability, pseudoparalysis, and cuff tear arthropathy. Some recent studies reported that the clinical effectiveness of exercise therapy and PT was equivalent to that of arthroscopic rotator cuff repair, whereas one recent study showed that the rotator cuff repair group had better clinical results than those in the nonsurgical group [8,9,10,11,12]. Thus, it remains unclear whether operative or conservative treatment is appropriate for every patient with ARCT.

**Figure 1:**
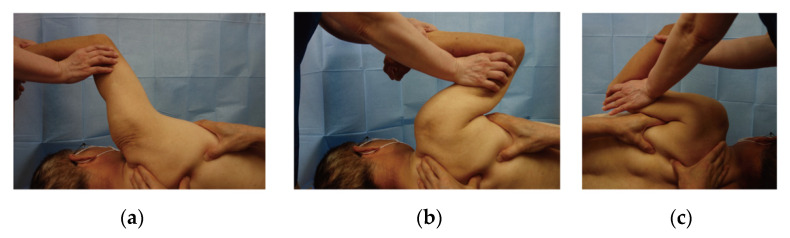
Adduction test of the glenohumeral joint (**a**) Starting position of adduction test, (**b**) Positive adduction test, (**c**) Negative adduction test.

The surgical procedure of arthroscopic rotator cuff repair implies that one pathology appears to convert asymptomatic tears into symptomatic tears. The connective tissues surrounding the supraspinatus tendon, including the upper capsule, coracohumeral ligament (CHL), and subacromial bursa, are dissected before tendon repair to evaluate torn tendon elasticity and determine a site of suturing to the bone. The dissection, which eliminates stiffness at the upper portion of the glenohumeral joint (GHJ), may suggest one pathology for symptomatic ARCT [13,14]. From this point of view, we investigated and reported the adduction restriction of the GHJ in patients with ARCTs as a new pathology of shoulder stiffness [15]. The restriction was identified in 70% of patients with symptomatic ARCTs using an adduction test (Figure 1), and the percentage of positive adduction tests was higher in smaller tears compared with larger tears. Furthermore, adduction manipulation (AM) of the GHJ provided excellent outcomes in a short period of time [15]. A recent study comparing manual therapy and/or exercise for the treatment of people with rotator cuff tears found no clinical differences between treatment groups [16]. A recent randomized study stated that following progressive exercise and best practice advice, without or with corticosteroid injections, did not result in significant differences in pain and functional activities after 12 months of treatment [17]. Given that the AM procedure provides better clinical results than PT, the new procedure would enhance the effectiveness of conservative treatment for ARCTs. The present study aimed to determine whether AM or PT was the best treatment option for ARCTs. We hypothesized AM would have higher treatment effectiveness than PT.

(a) Starting position of adduction test

The subject being tested lay in the lateral decubitus position on an examination table. One examiner abducted the humerus up to 100–120°. The upward rotated scapula was fixed with two hands by another examiner.

(b) Positive adduction test

When the upper arm was pushed gently toward the side in the coronal plane and the upper arm did not touch the side due to pain, the adduction test was positive.

(c) Negative adduction test

When the upper arm easily touched the side, the test was negative (no adduction restriction of the glenohumeral joint).

## 2. Materials and Methods

### 2.1. Patient Enrollment

**Figure 2:**
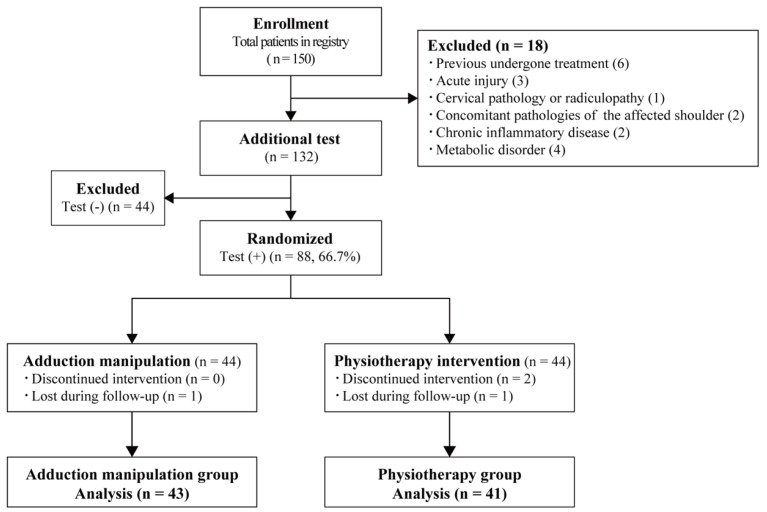
Flow diagram describing patients’ enrollment, allocation, adduction test, and grouping.

We obtained permission from the Institutional Review Board of the authors’ institute (K-2017-04) to conduct the present study, registering the study design and expected results in the UMIN Clinical Trials Registry (UMIN000045080, 6 August 2021). We enrolled 150 patients in our hospital who had shoulder pain and dysfunction and were identified as having rotator cuff tears using magnetic resonance imaging (MRI; Echelon RX, 1.5T; Hitachi, Tokyo, Japan). The shoulder surgeon measured the tear sizes using MRI and divided them into partial, small, medium, large, and massive in accordance with Cofield’s classification [18]. The inclusion criteria for patients with symptomatic ARCTs were duration of symptoms <6 months, normal findings in shoulder X-rays, and the identification of rotator cuff tears by MRI. Patients with trauma within the last 6 months, previous treatment in other hospitals, nerve compression in the neck, accompanying shoulder abnormalities in the same side shoulder, and systemic disorders were excluded from this study. Eighteen patients who had undergone previous treatment were excluded from this study (*n* = 6), as were those with acute injuries (*n* = 3), cervical myelopathy (*n* = 1), acromioclavicular osteoarthritis (*n* = 1), scapular dyskinesia (*n* = 1), chronic inflammatory disease (*n* = 2), and diabetic mellitus (*n* = 4) (Figure 2). The adduction test was performed in 132 patients, and 88 patients (66.7%) showed a positive adduction test and were included in this study, whereas the 44 patients (33.3%) with a negative adduction test were excluded (Figure 2).

### 2.2. Randomization

We determined that sex and age were the covariates, dividing patients into two groups using covariate adaptive randomization. Additionally, we used sequentially numbered, opaque, and sealed envelopes containing the randomization result. Each envelope was sequentially opened by the recruiting physician after the patient’s acceptance of participation according to the number on the envelope. The participants were assigned to the AM or PT treatment group after an explanation of the defined covariates and first assignments at enrollment in the study. Based on the patient’s baseline characteristics, we adjusted the number of patients in both groups. Eighty-eight patients were randomly assigned to the AM group (*n* = 44) or the PT group (*n* = 44) (Figure 2). We explained the benefits and adverse events of both treatments to patients, obtaining patients’ informed consent to participate in the present study.

### 2.3. Physiotherapy Group

The 44 patients allocated to the PT group received structured PT twice a week, and three physical therapists who were specialists in shoulder disorders treated the patients. First, the rehabilitation program started with assessment and patient education; patients learned about rotator cuff tears, as well as their malposture and painful motion. The physical therapists measured the active and passive range of motion (ROM) of the bilateral shoulders; examined which muscles were tight and tender; investigated thorax movement including the thoracic spine, clavicle, and ribs; and evaluated passive scapular motion [19,20]. Next, based on the concept that movements of the bones (the spine, ribs, clavicle, scapular, and humerus) are essential to shoulder motion, patients’ bone movements were improved using the mobilization of the costovertebral, sternocostal, sternoclavicular, and acromioclavicular joints. Massage was needed to relax tight muscles in the shoulder complex, including the rotator cuff, deltoid, pectorals major and minor, latissimus dorsi, teres major, biceps and triceps brachii muscles. Physical therapists performed massage by hands perpendicular to the muscles’ fiber direction to relax them. After increasing in passive shoulder motion, active ROM exercises such as flexion, external rotation (ER) at the side, internal rotation (IR) toward the back, and horizontal flexion were started. Finally, an isometric strengthening exercise of the rotator cuff and deltoid muscles was initiated when every active ROM reached 80% of that in the contralateral shoulder and no pain was encountered in the activities of daily living.

The goals of treatment were set as a visual analog scale (VAS) of pain score <1.0 in the activities of daily living and an American Shoulder and Elbow Surgeons (ASES) score >90 points. Patients who reached these goals were discharged from the structured PT intervention, and they visited the hospital every month to determine whether pain were absent. The ROM was maintained up to the 12-month follow-up appointment. Patients who could not achieve the treatment goal within 12 months continued PT and were recommended for surgical treatment.

### 2.4. Adduction Manipulation Group

The 44 patients allocated to the AM group received the AM procedure within 2 weeks of the initial examination. The AM procedure was conducted only once during the treatment period on an outpatient basis (Figure 2). An AM procedure was performed with the same maneuver as the adduction test under local anesthesia. In a sitting position, 12 mL of 1% lidocaine was injected from the tip of the coracoid process into three sites in 4 mL doses each: the rotator interval, subacromial bursa, and glenohumeral joint. The patients were lain in the lateral decubitus position on an examination table. One physician abducted the shoulder joint up to 120° and another physician held the scapula rotated upwardly as Figure 1a. The upper arm was pushed in the coronal plane to touch the side as Figure 1c. The AM procedure was repeated three times [15]. After the AM procedure, the patients in the AM group had a PT intervention once a week with the physiotherapists to improve movements of the bones (e.g., the scapula, clavicle, and ribs) through mobilization of the costovertebral, sternocostal, sternoclavicular, and acromioclavicular joints. The goal of the treatment was set to follow the previous description in the PT group. Patients with reduced pain, restored ROM, and improved muscle strength did not undergo any treatment by physicians but visited the hospital every month during the 12-month follow-up period.

### 2.5. Outcome Assessments

Age, sex, dominant arm, and body mass index (BMI) were recorded as baseline characteristics and clinical items were evaluated at the initial examination and at 1, 3, 6, and 12 months. Passive ROM of bilateral shoulder joints (i.e., flexion, abduction, ER, and IR) was recorded in the standing position using a goniometer. The VAS pain score, ASES score, and Constant score were also recorded. The clinical effectiveness of the AM and PT interventions was evaluated.

### 2.6. Measurement of Radiographic Glenohumeral Adduction Angle

**Figure 3:**
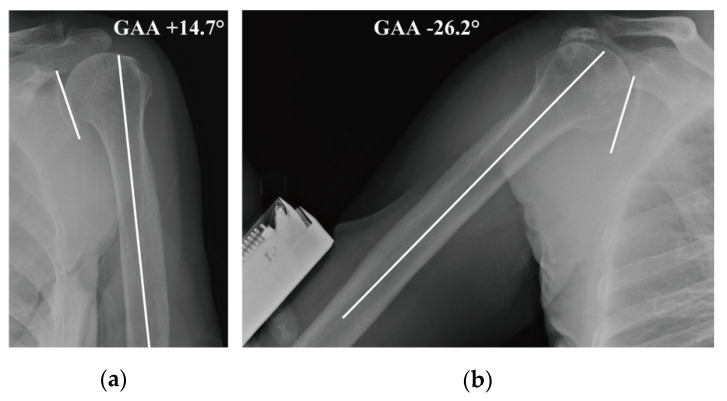
Radiographic glenohumeral adduction angle (**a**) Glenohumeral adduction angle (GAA) in a normal subject (**b**) GAA in a patient with rotator cuff tear and adduction restriction.

At the initial and final visits, the radiographic glenohumeral adduction angle (GAA) was calculated using a previously reported method [15]. The adduction of the GHJ was designated “plus”, the parallel of the glenoid line and humeral line was designated “zero”, and the abduction of the GHJ was designated “minus” (Figure 3a,b).

(a) Glenohumeral adduction angle (GAA) in a normal subject

The GAA in a normal subject showed 14.7° of adduction in the glenohumeral joint.

(b) GAA in a patient with rotator cuff tear and adduction restriction

The GAA was −26.2° and this meant 26.2° of abduction in the glenohumeral joint.

### 2.7. Statistics

Data are described as mean (95% confidence interval [CI]) or median (interquartile range [IQR]) values. A EZR on R commander version 1.52, a free statistical software that supports many functions, was utilized for statistical analysis [21]. Student’s *t* test was performed to compare age, BMI, arm dominance, symptoms, and treatment durations between groups. Percentages of sex, affected side, and tear size between treatment groups were compared using the chi-squared test. Two-way repeated-measures analysis of variance with group × follow-up periods was performed to assess improvement in clinical outcomes, except for IR, and Student’s *t* test was used to clarify intergroup differences at baseline and at 1, 3, 6, and 12 months thereafter. Additionally, IR in the two groups was compared using the Mann–Whitney test. All tests were conducted as two-sided tests, and *p* < 0.05 was considered to represent statistical significance.

## 3. Results

Two patients did not continue PT treatment, one patient in the PT group dropped out of the study during the follow-up period, and one patient in the AM group dropped out of the study. Thus, the final analysis included 43 patients in the AM group and 41 in the PT group (Figure 2). The patients’ background characteristics are summarized in Table 1. Age, sex, BMI, dominance of the affected shoulder, and mean symptom duration in both groups did not significantly differ between groups. Additionally, the numbers of tear sizes in the two groups did not differ significantly (*p* = 0.98). The mean treatment duration of the AM group was 3.3 months (95% CI: 2.5 to 4.1 months), which was much shorter than the 6.1 months in the PT group (95% CI: 4.3 to 7.9; *p* < 0.01).

**Figure 4:**
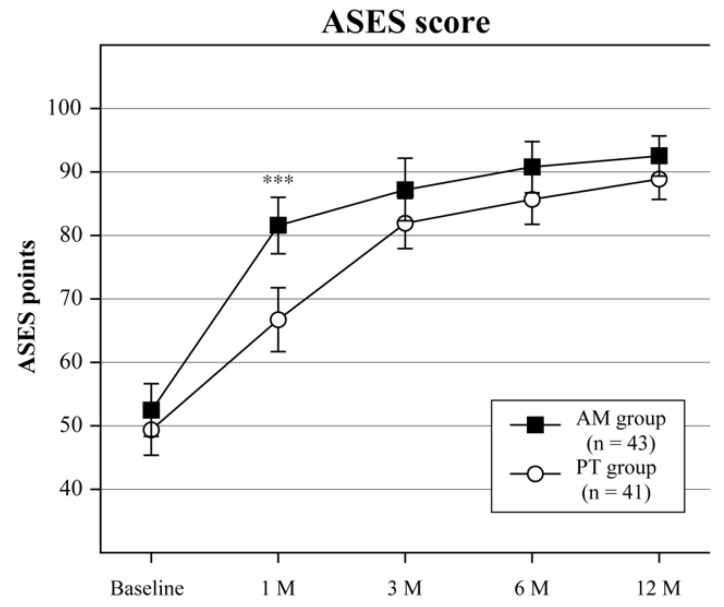
American Shoulder and Elbow Surgeons score, *** *p* < 0.001, AM—adduction manipulation group, PT–physiotherapy group.

**Figure 5:**
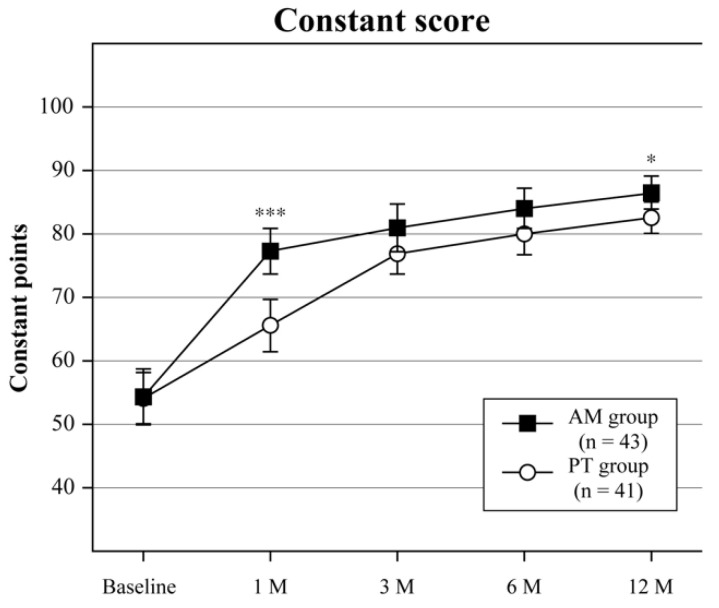
Constant score. Plot showing Constant scores at baseline and all follow-ups for the adduction manipulation (AM) group and the physiotherapy (PT) group. The error bars indicate 95% CIs. * *p* < 0.05, *** *p* < 0.001.

**Figure 6:**
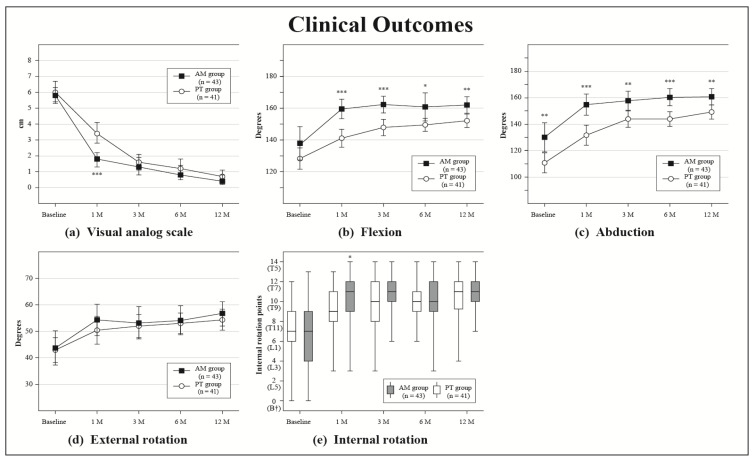
Clinical outcomes of pain and ROM. Plot showing results for visual analog scale (**a**); flexion (**b**); abduction (**c**); external ration (**d**); internal rotation (**e**) at baseline and all follow-ups for the adduction manipulation (AM) group and the physiotherapy (PT) group. The error bars indicate 95% CIs. * *p* < 0.05, ** *p* < 0.01, *** *p* < 0.001; T in (**e**), thoracic spine; L, lumber spine; B†, buttock.

Baseline and follow-up clinical outcome measurements are shown in Table 2. All clinical outcomes at 1 month in the AM group were greatly improved compared with those at baseline, but further significant changes were not found after 3 months. In the PT group, the VAS, ROM, ASES, and Constant scores gradually improved up to 3 months. A comparison between clinical outcomes at baseline and 12 months indicated a significant improvement in both groups. Clinical outcomes after 1 month were better in the AM group than in the PT group for VAS (*p* < 0.001), flexion (*p* < 0.001), abduction (*p* < 0.001), ER (*p* = 0.319), IR (*p* < 0.05), ASES score (*p* < 0.001), and Constant score (*p* < 0.001) (Table 2, Figure 4, Figure 5 and Figure 6). The final outcomes for flexion (*p* < 0.01), abduction (*p* < 0.01), and Constant score (*p* < 0.05) were also greater in the AM group than in the PT group (Table 2 and Figure 4, Figure 5 and Figure 6).

Plot showing American Shoulder and Elbow Surgeons (ASES) scores at baseline and all follow-ups for the adduction manipulation (AM) group and the physiotherapy (PT) group. The error bars indicate 95% CIs. *** *p* < 0.001.

The mean GAA at the initial visit was −21.1° in the PT group and −21.6° in the AM group (*p* = 0.738), and the angle at the 12-month follow-up decreased to −14.4° in the PT group and −3.2° in the AM group (*p* < 0.001) (Table 2). The mean GAA in the 44 patients who had a negative adduction test and were excluded from the present study was 2.1°, which was larger than those observed in both treatment groups (*p* < 0.001). All patients in the AM group had negative adduction tests and 16 patients (39%) in the PT group had positive tests at the 12-month follow-up. Eleven patients (13%) had unsatisfactory outcomes and transitioned to surgery after follow-up: 5 patients (11.6%) in the AM group (4 patients with arthroscopic rotator cuff repair and 1 with reverse shoulder arthroplasty) and 6 (14.6%) in the PT group (all with arthroscopic rotator cuff repair).

## 4. Discussion

The important findings of the present study were that (1) the AM procedure achieved pain relief and improved functional ability in patients with adduction restriction after a short period compared with the PT group; (2) flexion, abduction, ASES and Constant scores of the AM group were better than those of the PT group at the final evaluation; (3) and 16 patients in the PT group still demonstrated positive adduction tests after PT treatment, as well as a negative GAA. The AM procedure is a favorable treatment option for patients with adduction restriction. Efficacy was achieved in a short period of time and maintained at 12 months after the procedure. PT intervention without the AM procedure was unable to completely eliminate the adduction restriction, despite the full removal of the restriction using the AM procedure. PT or exercise therapy has been recognized as the first conservative treatment option for ARCTs [2,22,23]; however, the AM procedure is recommended as an initial conservative treatment before PT for patients with ARCTs with concomitant adduction restriction.

Our previous study demonstrated that the GAA in healthy individuals is 4.8° (i.e., 4.8° of adduction in the GHJ) and that a decrease in this angle with aging is likely to result in adduction restriction [15]. The GAA at the initial visit in both groups was −21°, and the GAA of the AM group at the final examination was −3.2°, compared with −14.4° in the PT group; therefore, the AM procedure eliminated adduction restriction. Our findings indicate that the effectiveness of PT for use in adduction restriction is less than that of AM. Radiological adduction restriction implies a shortening of the distance between the attachment of the supraspinatus and the superior rim of the glenoid. The tightness of the supraspinatus tendon and muscle, thickening of the upper capsule and CHL, and adhesion of the bursa due to inflammation or mechanical stress may cause the adduction restriction. As shown by microscopic examination, the CHL encloses both the supraspinatus and infraspinatus, and the joint capsule covers its intra-articular side [24,25]. The ligament originating from the coracoid process covers the outside of the supraspinatus and infraspinatus tendons, extends through the rotator interval, and spreads the supraspinatus and infraspinatus tendon on the joint side. The thickening of the bursal and articular sides of the ligament may be a cause of the adduction restriction. One MRI study demonstrated the formation of neovascular vessels and an increase in abnormal blood flow at the rotator interval in ARCTs [26]. The increased blood flow induces the inflammation and thickening of the CHL, upper capsule, and bursa. Additionally, the muscle tightness of the supraspinatus may also lead to adduction restriction. One study showed that stiffness of the supraspinatus musculotendinous unit was increased with partial and small tears but not in medium and large tears [27]. This finding is consistent with the result in our previous study, which showed that a positive adduction test is frequently identified in patients with partial or smaller tears compared to those with large tears [15]. An improvement in the relaxation of the supraspinatus muscle through PT may eliminate adduction restriction, whereas patients with a thickening of the CHL may continue to have adduction restriction.

The pathophysiology of symptomatic ARCTs is not fully understood despite many publications regarding the disorder. ARCTs are associated with abnormal shoulder and thoracic posture, joint contracture, weakness of the rotator cuff and scapular muscles, synovial inflammation in the GHJ, subacromial impingement, joint contracture, instability, pseudoparalysis, and cuff tear arthropathy [4,5,6,7,28,29]. The administration of NSAIDs and an injection of corticosteroids into the joint and/or bursa decreases inflammation in the tissues. PT and exercise therapy are able to improve kinematic problems including malposture, joint contracture, atrophy of the rotator cuff and scapular muscles, and scapular motion. These treatment options are standard conservative treatments but the AM procedure for the adduction restriction of the GHJ has not been included as a non-surgical treatment option [12]. Patients who have persisting pain and/or disability following conservative treatment, including muscle weakness, instability, pseudoparalysis, and cuff tear arthropathy, have to undergo surgical treatment such as arthroscopic rotator cuff repair, superior capsular reconstruction, or reverse shoulder arthroplasty. However, treatment options for ARCTs are sometimes tailored individually. Pain severity, diverse symptoms, and physical function, as well as the patient’s needs, tear type and size, and disability in daily living, have to be considered when physicians determine the appropriate treatment. An interesting investigation suggested that patients who have lower expectations of PT treatment, who need higher activity levels in daily life and sport, and who affirm smoking are likely to undergo surgery, regardless of their symptoms and the anatomical findings of rotator cuff tears [30]. The principle that conservative management, including PT and corticosteroid injections, is the first treatment option is essential; furthermore, the AM procedure ensures the immediate restoration of shoulder function and pain relief for patients with ARCTs.

Rehabilitation programs include correcting malposture; increasing movement of the thoracic spine, ribs, clavicle, and scapula; removing joint contracture; and strengthening the deltoid, rotator cuff, and periscapular muscles [22,23]. Recent studies have reported that PT provides equivalent outcomes to surgical repair [9,10,11,31,32]. One study stated that a specific PT for 3 months was effective in 75% of patients [2]. This duration of PT treatment is consistent with our findings that the VAS, ROM, ASES, and Constant scores gradually recovered up to 3 months. The systematic review summarized the effectiveness of PT for treating complete rotator cuff tears [22]. Exercise and rehabilitation programs show considerable variation across the techniques among studies: muscle strengthening was performed in more than 90% of the studies; ROM exercises in 80%; muscle stretching, joint mobility, and activity modification and advice in 60%; home exercise routine in 32%; and postural intervention and manual therapy in 20% [22]. The review demonstrated that many exercise regimens and rehabilitation programs are used for patients with rotator cuff tears, which means PT and exercise programs vary in every hospital. In the present study, clinical results in the AM group were better than those in the PT group; therefore, the AM procedure is more beneficial and should be conducted before formal PT or exercise therapy treatment. Thirteen percent of patients in both groups who needed surgical management in the present study showed muscle weakness, instability, and pseudoparalysis. The percentage of patients who required surgery in our study is similar to that in a recent study, in which 15% of patients underwent surgery by the 2-year follow-up appointment [2]. Although the AM procedure is a useful treatment tool for patients, surgical treatment is still needed for 10 to 20% of patients with ARCTs. As the tear size of full-thickness rotator cuff tears is likely to progress 3.8 mm in length and 2.0 mm in width per year [33,34], physicians have to pay attention to patients’ shoulder conditions after AM procedures.

The present study has some limitations, the main one being that the pathology underlying adduction restriction has not been fully delineated. Therefore, we need to macroscopically and microscopically examine the upper part of the joint and subacromial bursa using cadavers with and without ARCTs. Other limitations include that the number of patients in both groups was less than 50 and that the duration of follow-up was 1 year. A similar study with more than 100 patients in each group and a 2-year follow-up would be more appropriate. Another limitation is that the number of massive and large tears was too small in both groups to compare clinical results. Adduction tests and manipulation should be conducted in patients with large and massive tears to compare the clinical efficiencies of PT and AM in the future. The final limitation of this study is that we did not investigate patient-reported outcome measures, such as by feedback using the 36-item short-form survey (SF-36). Recent research has recommended the adoption of a quantitative approach to measuring aspects of health status by asking patients directly for their opinion using a standardized questionnaire.

## 5. Conclusions

In the present study, we demonstrated that adduction restriction of the GHJ is concomitant in 67% of patients with ARCTs. The AM procedure was more effective than PT at every follow-up timepoint. The AM procedure was able to completely eliminate the restriction, whereas PT only partly removed the restriction; therefore, it is recommended that researchers use the AM procedure as a first conservative treatment option over PT in order to achieve favorable clinical outcomes.

## Figures and Tables

**Table 1:** Patient characteristics of 2 treatment groups.

	AM Group (*n* = 43)	PT Group (*n* = 41)	*p* Value
Age, yr	71.3(69.0, 73.6) *	70.7 (67.8, 73.6) *	0.744
Sex, male, *n* (%)	23 (53.5%)	16 (39.0%)	0.198
female, *n* (%)	20 (46.5%)	25 (61.0%)	
BMI (kg/m^2^)	24.0 (23.1, 24.9) *	24.4 (23.6, 25.2) *	0.516
Side, right (%)	36 (83.7%)	24 (58.5%)	0.015
left (%)	7 (16.3%)	17 (41.5%)	
Dominance, right (%)	39 (90.7%)	39 (95.1%)	0.676
left (%)	4 (9.3%)	2 (4.9%)	
Symptom duration (M)	3.2 (2.8, 3.7) *	2.7 (2.3, 3.1) *	0.115
Treatment duration (M)	3.3 (2.5, 4.1) *	6.1 (4.3, 7.9) *	0.004
Tear size *n* (%)			
Massive	3 (7.0%)	2 (4.9%)	0.983
Large	5 (11.6%)	6 (14.6%)	
Medium	17 (39.5%)	15 (36.6%)	
Small	13 (30.2%)	14 (34.1%)	
Partial	5 (11.6%)	4 (9.8%)	

* Values are shown as the mean (95% CI); AM group, adduction manipulation group; PT, physiotherapy group; BMI, body mass index; M, month.

**Table 2:** Clinical outcomes measures from a 2-Way ANOVA between 2 treatment groups.

	AM Group	PT Group	Tow-Way ANOVA		*t* Test
	Mean (95%CI)	Mean (95%CI)	*F* Value	*p* Value	*p* Value
GAA			99.8	<0.001	
Baseline	−21.6 (−19.8–−23.3)	−21.1 (−19.6–−24.2)			0.738
12 Months	−3.2 (−1.4–−5.0)	−14.4 (−11.9–−16.9)			<0.001
VAS			4.956	<0.001	
Baseline	5.8 (5.3–6.3)	6.0 (5.4–6.7)			0.473
1 Month	1.8 (1.3–2.2)	3.4 (2.8–4.1)			<0.001
3 Months	1.3 (0.8–1.9)	1.6 (1.1–2.1)			0.471
6 Months	0.9 (0.5–1.4)	1.2 (0.7–1.8)			0.455
12 Months	0.5 (0.2–0.8)	0.7 (0.3–1.1)			0.406
Flexion			1.605	0.173	
Baseline	137.9 (127.4–148.4)	128.3 (121.6–135.0)			0.127
1 Month	159.5 (153.4–165.7)	141.1 (135.4–146.8)			<0.001
3 Months	162.3 (157.0–167.5)	147.9 (142.7–153.0)			<0.001
6 Months	160.8 (152.0–169.6)	149.5 (145.4–153.6)			<0.05
12 Months	162.0 (156.8–167.2)	152.1 (147.9–156.3)			<0.01
Abduction			1.604	0.173	
Baseline	130.0 (119.0–141.0)	110.7 (103.2–118.3)			<0.01
1 Month	154.7 (146.7–162.6)	131.6 (124.0–139.1)			<0.001
3 Months	157.7 (150.5–164.9)	143.9 (137.7–150.0)			<0.01
6 Months	160.2 (153.8–166.7)	143.8 (138.2–149.3)			<0.001
12 Months	160.6 (154.4–166.8)	149.1 (143.7–154.5)			<0.01
ER			0.682	0.605	
Baseline	43.7 (37.3–50.2)	42.9 (38.2–47.6)			0.842
1 Month	54.3 (48.4–60.2)	50.4 (45.1–55.6)			0.319
3 Months	53.2 (47.1–59.3)	52.0 (47.6–56.4)			0.748
6 Months	54.1 (48.7–59.6)	53.0 (49.1–56.9)			0.732
12 Months	56.8 (52.0–61.2)	54.3 (50.4–58.3)			0.434
ASES			5.877	<0.001	
Baseline	52.5 (48.3–56.6)	49.4 (45.4–53.4)			0.282
1 Month	81.6 (77.1–86.0)	66.7 (61.7–71.7)			<0.001
3 Months	87.2 (82.3–92.1)	81.9 (77.9–85.8)			0.095
6 Months	90.8 (86.7–94.8)	85.7 (81.8–89.6)			0.075
12 Months	92.6 (89.4–95.7)	88.9 (85.7–92.0)			0.096
Constant			6.870	<0.001	
Baseline	54.3 (49.9–58.7)	54.1 (50.1–58.2)			0.952
1 Month	77.3 (73.7–80.9)	65.6 (61.4–69.7)			<0.001
3 Months	81.0 (77.2–84.7)	76.9 (73.7–80.2)			0.108
6 Months	84.0 (80.9–87.2)	80.0 (76.7–83.2)			0.069
12 Months	86.5 (83.9–89.1)	82.6 (80.1–85.1)			<0.05
	median (IQR)	median (IQR)			U test
IR					
Baseline	T12 (L3–T10)	T12 (L1–T10)			0.613
1 Month	T8 (T10–T7)	T10 (T11–T8)			0.05
3 Months	T8 (T9–T7)	T9 (T11–T7)			0.216
6 Months	T9 (T10–T7)	T9 (T10–T8)			0.496
12 Months	T8 (T9–T7)	T8 (T10–T7)			0.342

AM group, adduction manipulation group; PT, physiotherapy group; GAA, glenohumeral adduction angle; VAS, visual analog scale; ER, external rotation at the side; ASES, American Shoulder and Elbow Surgeons; U test, Mann–Whitney U test; IR, internal rotation at the back; IQR, interquartile range; T; thoracic spine, L; lumber spine.

## Data Availability

We registered the study UMIN Clinical Trials Registry (UMIN000045080, 6 August 2021).

## References

[1] Yamamoto A., Takagishi K., Osawa T., Yangawa T., Nakajima D., Shitara H., Kobayashi T. (2010). Prevalence and Risk Factors of a Rotator Cuff Tear in the General Population. J. Shoulder Elbow Surg..

[2] Kuhn J.E., Dunn W.R., Sanders R., An Q., Baumgarten K.M., Bishop J.Y., Brophy R.H., Carey J.L., Holloway B.G., Jones G.L. (2013). Effectiveness of Physical Therapy in Treating Atraumatic Full-Thickness Rotator Cuff Tears: A Multicenter Prospective Cohort Study. J. Shoulder Elbow Surg..

[3] Minagawa H., Yamamoto N., Abe H., Fukuda M., Seki N., Kikuchi K., Kijima H., Itoi E. (2013). Prevalence of Symptomatic and Asymptomatic Rotator Cuff Tears in the General Population: From Mass-Screening in One Village. J. Orthop..

[4] Seo S.S., Choi J.S., An K.C., Kim J.H., Kim S.B. (2012). The Factors Affecting Stiffness Occurring with Rotator Cuff Tear. J. Shoulder Elbow Surg..

[5] Tauro J.C. (2006). Stiffness and Rotator Cuff Tears: Incidence, Arthroscopic Findings, and Treatment Results. Arthroscopy.

[6] Collin N.P., Matsumura A., Lädermann P., Denard J., Walch G. (2014). Relationship between Massive Chronic Rotator Cuff Tear Pattern and Loss of Active Shoulder Range of Motion. J. Shoulder Elbow Surg..

[7] Hölscher T., Weber T., Lazarev I., Englert C., Dendorfer S. (2016). Influence of Rotator Cuff Tears on Glenohumeral Stability during Abduction Tasks. J. Orthop. Res..

[8] Kukkonen J., Joukainen A., Lehtinen J., Mattila K.T., Tuominen E.K.J., Kauko T., Aärimaa V. (2014). Treatment of Non-Traumatic Rotator Cuff Tears: A Randomized Controlled Trial with One-Year Clinical Results. Bone Jt. J..

[9] Moosmayer S., Lund G., Seljom U.S., Haldorsen B., Svege I.C., Hennig T., Pripp A.H., Smith H. (2014). Tendon Repair Compared with Physiotherapy in the Treatment of Rotator Cuff Tears: A Randomized Controlled Study in 103 Cases with A Five-Year Follow-Up. J. Bone Jt. Surg. Am..

[10] Kukkonen J., Joukainen A., Lehtinen J., Mattila K.T., Tuominen E.K., Kauko T., Äärimaa V. (2015). Treatment of Nontraumatic Rotator Cuff Tears: A Randomized Controlled Trial with Two Years of Clinical and Imaging Follow-Up. J. Bone Jt. Surg. Am..

[11] Lambers Heerspink F.O., van Raay J.J., Koorevaar R.C., van Eerden P.J., Westerbeek R.E., van’t Riet E., van den Akker-Scheek I., Diercks R.L. (2015). Comparing Surgical Repair with Conservative Treatment for Degenerative Rotator Cuff Tears: A Randomized Controlled Trial. J. Shoulder Elbow Surg..

[12] Ramme A.J., Robbins C.B., Patel K.A., Carpenter J.E., Bedi A., Gagnier J.J., Miller B.S. (2019). Surgical Versus Nonsurgical Management of Rotator Cuff Tears. J. Bone Jt. Surg. Am..

[13] Hatakeyama Y., Itoi E., Urayama M., Pradhan R.L., Sato K. (2001). Effect of Superior Capsule and Coracohumeral Ligament Release on Strain in The Repaired Rotator Cuff Tendon. A Cadaveric Study. Am. J. Sports Med..

[14] Park J.H., Yang S.H., Rhee S.M., Oh J.H. (2019). The Effect of Concomitant Coracohumeral Ligament Release in Arthroscopic Rotator Cuff Repair to Prevent Postoperative Stiffness: A Retrospective Comparative Study. Knee Surg. Sports Traumatol. Arthrosc..

[15] Yano Y., Hamada J., Hagiwara Y., Karasuno K.H., Tamai K., Suzuki K. (2020). A New Pathophysiology of Atraumatic Rotator Cuff Tears: Adduction Restriction of the Glenohumeral Joint. JSES Int..

[16] Page M.J., Green S., McBain B., Surace S.J., Deitch J., Lyttle N., Mrocki M.A., Buchbinder R. (2016). Electrotherapy Modalities for Rotatoe Cuff disease. Cochrane Database Syst. Rev..

[17] Hopewell S., Keene D.J., Marian I.R., Dritsaki M., Heine P., Cureton L., Dutton S.J., Dakin H., Carr A., Hamilton W. (2021). Progressive Exercise Compared with Best Practice Advice, with or without Corticosteroid Injection, for the Treatment of Patients with Rotator Cuff Disorders (GRASP): A Multicentre, Pragmatic, 2 × 2 Factorial, Randomised Controlled Trial. Lancet.

[18] Cofield R.H., Parvizi J., Hoffmeyer P.J., Lanzer W.L., Ilstrup D.M., Rowland C.M. (2001). Surgical Repair of Chronic Rotator Cuff Tears. A Prospective Long-Term Study. J. Bone Jt. Surg. Am..

[19] Tachihara H., Hamada J. (2019). Characteristic Movement of the Ribs, Thoracic Vertebrae and Scapula While Elevating the Upper Limbs-Influences of Age and Gender on Movements. Open Orthop. J..

[20] Endo K., Hamada J., Suzuki K., Hagiwara Y., Muraki T., Karasuno H. (2016). Does Scapular Motion Regress with Aging and Is It Restricted in Patients with Idiopathic Frozen Shoulder?. Open Orthop. J..

[21] Kanda Y. (2013). Investigation of the Freely Available Easy-To-Use Software ‘EZR’ for Medical Statistics. Bone Marrow Transplant..

[22] Jeanfavre M., Husted S., Leff G. (2018). Exercise Therapy in the Non-Operative Treatment of Full-Thickness Rotator Cuff Tears: A Systematic Review. Int. J. Sports Phys. Ther..

[23] Edwards P., Ebert J., Joss B., Bhabra G., Ackland T., Wang A. (2016). Exercise Rehabilitation in the Non-Operative Management of Rotator Cuff Tears: A Review of the Literature. Int. J. Sports Phys. Ther..

[24] Clark J.M., Harryman D.T. (1992). Tendons, Ligaments, and Capsule of the Rotator Cuff. Gross and Microscopic Anatomy. J. Bone Jt. Surg. Am..

[25] Arai R., Nimura A., Yamaguchi K., Yoshimura H., Sugaya H., Saji T., Mutsuda S., Akita K. (2014). The Anatomy of the Coracohumeral Ligament and Its Relation to the Subscapularis Muscle. J. Shoulder Elbow Surg..

[26] Sasanuma H., Sugimoto H., Iijima Y., Kanaya Y., Saito T., Takeshita K. (2018). Blood Flow Evaluation by Dynamic Magnetic Resonance Imaging of Symptomatic Rotator Cuff Tears and Frozen Shoulders. J. Shoulder Elbow Surg..

[27] Itoigawa Y., Wada T., Kawasaki T., Morikawa D., Maruyama Y., Kaneko K. (2020). Supraspinatus Muscle and Tendon Stiffness Changes After Arthroscopic Rotator Cuff Repair: A Shear Wave Elastography Assessment. J. Orthop. Res..

[28] Yamamoto A., Takagishi K., Kobayashi T., Shirata H., Ichinose T., Tkasawa E., Shimoyama D., Osawa T. (2015). The Impact of Faulty Posture on Rotator Cuff Tears with and without Symptoms. J. Shoulder Elbow Surg..

[29] Candela V., Aimino R., Mezzaqui L., Standoli J.P., Gumina S. (2022). Macroscopic Aspects of Glenohumeral Synovitis Are Related to Rotator Cuff Tear Severity. J. Shoulder Elbow Surg..

[30] Dunn W.R., Kuhn J.E., Sanders R., An Q., Baumgarten K.M., Bishop J.Y., Brophy R.H., Carey J.L., Harrell F., Holloway B.G. (2016). 2013 Neer Award: Predictors of Failure of Nonoperative Treatment of Chronic, Symptomatic, Full-Thickness Rotator Cuff Tears. J. Shoulder Elbow Surg..

[31] Ranebo M.C., Björnsson Hallgren H.C., Holmgren T., Adolfsson L.E. (2020). Surgery and Physiotherapy were Both Successful in the Treatment of Small, Acute, Traumatic Rotator Cuff Tears: A Prospective Randomized Trial. J. Shoulder Elbow Surg..

[32] Kukkonen J., Ryösä A., Joukainen A., Lehtinen J., Kauko T., Mattila K., Äärimaa V. (2021). Operative Versus Conservative Treatment of Small Non-Traumatic Supraspinatus Tears in Patients over 55 Years: Over 5-Year Follow-Up of a Randomized Controlled Trial. J. Shoulder Elbow Surg..

[33] Yamamoto N., Mineta M., Kawakami J., Sano H., Itoi E. (2017). Risk Factors for Tear Progression in Symptomatic Rotator Cuff Tears: A Prospective Study of 174 Shoulders. Am. J. Sports Med..

[34] Jung W., Lee S., Kim S.H. (2020). The Natural Course and Risk Factors for Tear Progression in Conservatively Treated Full-Thickness Rotator Cuff Tears. J. Shoulder Elbow Surg..

